# Polymorphism rs2395655 affects LEDGF/p75 binding activity and p21WAF1/CIP1 gene expression in esophageal squamous cell carcinoma

**DOI:** 10.1002/cam4.2067

**Published:** 2019-03-10

**Authors:** Rong Guo, Yunan Ma, Min Zhao, Wenlong Zhang, Guo An, Baojun Chen, Yiping Song, Hui Xu, Yong Li

**Affiliations:** ^1^ Department of Medical Oncology Cancer Hospital Chinese Academy of Medical Sciences & Peking Union Medical College Beijing China; ^2^ Key Laboratory of Carcinogenesis and Translational Research (Ministry of Education) Department of Laboratory Animal Peking University Cancer Hospital & Institute Beijing China; ^3^ Key Laboratory of Carcinogenesis and Translational Research (Ministry of Education) Department of Pathology Peking University Cancer Hospital & Institute Beijing China

**Keywords:** esophageal squamous cell carcinoma, lens epithelium‐derived growth factor, p21WAF1/CIP1, prognostic factor, single‐nucleotide polymorphism

## Abstract

p21WAF1/CIP1 (p21) plays critical roles in cell‐cycle regulation and DNA repair and is transcriptionally regulated through p53‐dependent or ‐independent pathways. Bioinformatic analysis predicated one stress‐response element (STRE) implicated in single nucleotide polymorphism (SNP) rs2395655 of the p21 promoter. Here, we investigated the transcriptional regulatory function of rs2395655 variant genotype and analyzed its associations with the p21 expression and clinical outcomes in esophageal squamous cell carcinoma (ESCC) patients. Luciferase assay results showed significantly increased transcriptional activity of the rs2395655 G allele‐containing p21 promoter compared with rs2395655 A allele‐containing counterpart, especially in ESCC cells with ectopic LEDGF/p75 expression. Furthermore electrophoretic mobility shift assay using the rs2395655 G or A allele‐containing probe and chromatin immunoprecipitation assay with specific anti‐LEDGF/p75 antibody indicated the potential binding activity of LEDGF/p75 with the STRE element implicated in rs2395655 G allele of the p21 promoter. Subsequent specific RNA interference‐mediated depletion or ectopic expression of LEDGF/p75 caused obviously down‐ or up‐regulated expression of p21 mRNA in ESCC cells harboring rs2395655 GG genotype but not cells with rs2395655 AA genotype. Furthermore, rs2395655 GG genotype carriers showed significantly elevated p21 protein expression and conferred survival advantage in both univariate and multivariate analyses in total 218 ESCC patients. Our findings suggest that LEDGF/p75 regulates the p21 expression in ESCC cells through interacting with STRE element implicated in polymorphism rs2395655 and the elevated p21 protein expression and rs2395655 GG genotype may serve as positive prognostic factors for ESCC patients.

## 1 INTRODUCTION

Esophageal cancer is one of the most common malignancies in the world. Esophageal squamous cell carcinoma (ESCC) constitutes 80% of esophageal cancer and the majority of ESCC cases occur in Asia, especially in north central China.^1^ In spite of recent advances in diagnosis and treatment, the survival rate has not been obviously improved,^2^ arousing the interest in search for additional indicators besides the conventional tumor‐node‐metastasis (TNM) staging system to better predict the outcome of ESCC patients.

p21WAF1/CIP1 (p21, AF 497972) is an essential mediator in the DNA damage response, by inducing cell cycle arrest, direct inhibition of DNA replication, as well as by regulating cell apoptosis. p21 interacts with cyclin/cyclin‐dependent kinase (CDK) complex and functions to negatively control cell cycle.^3^ p21 also directly binds with proliferating cell nuclear antigen (PCNA) and thereby inhibits DNA replication.^4^ Although p21 has been reported as a useful prognostic factor in a variety of human tumors, the clinicopathologic significance of the p21 expression for ESCC patients remains controversial.^5^, ^6^, ^7^, ^8^, ^9^, ^10^, ^11^, ^12^, ^13^

Transcriptional regulation is the key mechanism in control of the p21 expression. The activation of p21 promoter is regulated both by transcription factor p53 in response to DNA damage and by extracellular growth stimuli in a p53‐independent mechanism and the p21 promoter harbors several *cis*‐elements corresponding to these external and internal factors.^14^, ^15^, ^16^ There are two p53‐responsive elements located within nucleotides –2282 to –2263 and –1391 to –1361, respectively. In addition, the proximal p21 promoter region between nucleotides –125 and –45 contains several GC‐rich Sp1 motifs and several binding sites for transcription factor E2F as well as three E boxes.^17^, ^18^ Moreover, studies have revealed Ap‐1, Ap‐2, c‐Jun, c‐Ets‐1, C/EBP alpha, and STATs sites distributed throughout the p21 promoter region and suggested that the p21 promoter may integrate different signals via different set of transcription factors and *cis*‐elements into cellular decisions leading to proliferation or cell cycle arrest.^16^, ^19^, ^20^, ^21^ Besides the transcriptional level, the p21 expression has also been reported to be negatively regulated by some kinds of microRNA at the posttranscriptional level, such as miR‐17 which directly targeted the 3’‐UTR of the p21 mRNA.^22^

In this study, we performed bioinformatic analysis using the TFSEARCH program (http://www.cbrc.jp/research/db/TFSEARCH.html) and predicted that one *cis*‐regulatory element [stress‐response element, STRE; (T/A)GGGG(A/T)]^23^ is created when the p21 promoter harbors a guanidine at nucleotide –809, that is single‐nucleotide polymorphism (SNP) rs2395655 (–809G/A). We further examined the effects of rs2395655 (–809G/A) and another common polymorphism rs3829963 (–2119C/A) on expression regulation of the p21 gene. The results of luciferase assay demonstrated that the p21 promoter harboring rs2395655 G allele exhibited significantly higher transcriptional activity than rs2395655 A allele‐containing counterpart. This prediction was proved by the use of electrophoresis mobility shift assay (EMSA) and chromatin immunoprecipitation (ChIP) assay which both showed the capability of the p21 promoter with rs2395655 G but not A allele for binding to lens epithelium‐derived growth factor/p75 (LEDGF/p75, also known as PSIP1 and DFS70 autoantigen), a potent survival oncoprotein involved in stress response, autoimmune disease, HIV replication, and cancer progression.^24^, ^25^, ^26^, ^27^ The regulatory effect of LEDGF/p75 on the p21 expression was further examined in ESCC cells with rs2395655 GG or AA genotype. Moreover, the association of rs2395655 variant genotype with the p21 protein expression and their prognostic values in ESCC patients were investigated.

## 2 MATERIALS AND METHODS

### 2.1 Expression vectors and RNA interference

To generate reporter plasmids containing the p21 promoter with nucleotide alterations at positions −2119 and −809, a p21 promoter sequence comprising nucleotides −2308 to +204 with –2119C/–809G was first cloned from genomic DNA using primer sets: 5′‐CGGGGTACCGTGGCTCTGATTGGCTTTCTG‐3′ (forward) and 5′‐GGAAGATCTGAAACACCTGTGAACGCAGCAC‐3′ (reverse). The polymerase chain reaction (PCR) product was ligated into pGL3‐Basic vector (Promega, Madison, WI) and the resultant reporter construct was used as template to generate three other constructs containing −2119A/−809G, −2119C/−809A, and −2119A/−809A haplotypes, respectively, by using site‐specific mutagenesis. Expression vector containing the full coding domain sequence of LEDGF/p75 gene was constructed using the pcDNA3.0 plasmid (Invitrogen, Carlsbad, CA). All constructs were restriction mapped and sequenced. Two small interference RNAs (siRNAs) targeting nucleotides 1428 to 1448 and nucleotides 1340 to 1360 of human LEDGF/p75 mRNA (NM_033222.3) were synthesized and named as si‐p75‐1 and ‐2, respectively.

### 2.2 Cell lines and transient transfections

Four kinds of human ESCC‐derived cell lines (EC‐109, KYSE150, KYSE410, and KYSE450) were kindly provided by Dr. Chen KN (Peking University Cancer Hospital & Institute, Beijing, China). Cells were cultured in RPMI 1640 medium supplemented with 10% FBS and 1% penicillin/streptomycin stock in a humidified atmosphere of 5% CO_2_ at 37°C. LEDGF/p75 expression vector and si‐p75 transfection were performed as described.^28^ Briefly, exponentially growing ESCC cells were seeded into 24‐well plates (5 × 10^4^ cells/well). The next day, 0.8 μg of expression vector or si‐p75 was mixed with 2 μL Lipofectamine 2000 (Invitrogen) plus 50 μL Opt‐MEM medium (Invitrogen) for 20 minutes and then added to cells. For ectopic LEDGF/p75 expression and siRNA transfection, the empty vector (pcDNA3.0) and mismatched siRNA were used as controls, respectively. After transfection for 24 hours, cellular proteins were isolated for further analysis.

### 2.3 Luciferase assay

EC‐109 and KYSE150 cells were seeded into 24‐well plates. Twenty‐four hours later, cells were cotransfected with 0.4 μg of one reporter construct containing a p21 promoter haplotype, 2 ng of pRL‐SV40, and 0.4 μg of LEDGF/p75 expression vector or empty vector (pcDNA3.0) using Lipofectamine 2000 (Invitrogen). Luciferase activity was analyzed 48 hr after transfection using the Dual‐Luciferase Reporter Assay System (Promega). The pRL‐SV40 co‐transfected with pGL3‐Basic vector and pcDNA3.0 empty vector was used as control. The value of each report construct was calculated as the mean ± SD from three independent experiments each performed in duplicate.

### 2.4 Electrophoretic mobility shift assay (EMSA)

Nuclear extracts were prepared from KYSE150 cells with NE‐PER Nuclear and Cytoplasmic Extraction Reagents according to the manufacturer's instructions (Pierce, Rockford, IL). Synthetic double‐stranded oligonucleotides 5′‐GCAACCACAGGGATTTCTTCTGTTC‐3′ and 5′‐GCAACCACAGGGGTTTCTTCTGTTC‐3′ corresponding respectively to rs2395655 G and A alleles were labeled with biotin and the binding reactions were performed using the LightShift Chemiluminescent EMSA Kit (Pierce). For each gel shift reaction, 20 fmol of biotin‐labeled probe was incubated with 4 μg of nuclear extracts for 20 minutes at room temperature in a 20 μL mixture containing 1 × binding buffer, 2.5% glycerol, 5 mmol/L MgCl_2_, 50 ng/μL Poly(dI:dC), and 0.05% NP‐40. The resulting protein‐DNA complexes were subjected to electrophoresis on a 6% native PAGE, transferred to a positively charged nylon membrane (GE Healthcare Life Sciences, Pittsburgh, PA), and then cross‐linked using a GS Gene Linker UV chamber (BioRad, Hercules, CA). To demonstrate the specificity of protein‐DNA complex formation, an anti‐LEDGF/p75 antibody (C‐16) (Santa Cruz, CA) or a 400‐fold molar excess of cold probe was added to the binding reaction. Biotin‐labeled DNA was detected using stabilized streptavidin/horseradish peroxidase conjugate (Pierce).

### 2.5 Chromatin immunoprecipitation (ChIP) assay

Chromatin immunoprecipitation was performed using EZ‐Magna ChIP TMA Chromatin Immunoprecipitation Kit (Millipore, Billerica, MA) following the manufacturer's instructions. Formaldehyde cross‐linked chromatin obtained from EC‐109 and KYSE410 cells was sonicated to generate DNA fragments from 200 bp to 1000 bp. Anti‐LEDGF/p75 antibody (C‐16) (Santa Cruz) was used to precipitate LEDGF/p75. ChIP sample (5% of input) was used as a positive control and IgG (Santa Cruz) as a negative control. Oligonucleotide primers used to amplify genomic DNA for ChIP are shown in Table 1. All immunoprecipitation assays and PCR amplifications were repeated with reproducible results.

**Table 1:** Primers for RT‐PCR analysis and ChIP assay

Genes	Primers, 5′‐ 3′	Annealing (°C)	Product (bp)
β*‐actin* (RT‐PCR)	Forward: GGAGAAAATCTGGCACCACAC Reverse: CGTACAGGTCTTTGCGGATGT	57	638
*LEDGF/p75* (RT‐PCR)	Forward: CACACAGAGATGATTACTACACTG Reverse: CCATCTTGAGCATCAGATCCTC	52	284
*P21* (RT‐PCR)	Forward: GCCTGCCGCCGCCTCTTC Reverse: GAATTCAGGTCTGAGTGTCCAGGA	57	888
*GAPDH* (ChIP)	Forward: ACATCGTGACCTTCCGTGC Reverse: GCTGAGAGGCGGGAAAGT	56	282
*P21* (ChIP and rs2395655 sequencing)	Forward: CTGCATGATCTGAGTTAGGTCAC Reverse: ACCCTACACTCACCTGAACAG	62	161
*FBXO10* (ChIP)	Forward: CTACAGCAGTGTCCTACAAGAGCA Reverse: GCCTAGTGGCTGGTACCTGTT	56	228

### 2.6 Western blot analysis

Cells were lysed on ice for 25 minutes in lysis buffer (15 mmol/L Tris‐HCl pH 7.5, 150 mmol/L NaCl, 0.1% Tween 20, and 1 mmol/L DTT) supplemented with protease inhibitors and the solution was cleared by centrifugation at 14 000 *g* for 25 minutes. The total protein concentration was quantified utilizing a BCA protein assay kit and equal amounts of proteins (10‐20 μg/lane) were separated on 10%‐12% SDS‐PAGE gels and electrotransferred to PVDF membranes. The membranes were then blocked with 5% nonfat milk and probed with specific primary antibodies: anti‐LEDGF/p75 (BD Biosciences, Heidelberg, Germany) or anti‐Actin (Santa Cruz) (1:1000 to 1:5000 dilution), respectively. After washing with 0.2% PBS‐T buffer four times, membranes were incubated with horseradish peroxidase‐conjugated secondary antibody and visualized using the enhanced chemiluminescence system, ECL (GE Healthcare Life Sciences).

### 2.7 Total RNA isolation and semi‐quantitative reverse transcription‐PCR (RT‐PCR)

Total cellular RNA was isolated with Trizol reagent (Life Technologies, Gaithersburg, MD) according to the manufacturer's instruction. Approximately 2 μg of total RNA from each cell line was digested with DNase I to remove DNA contamination and reverse transcribed into cDNA using the Superscript III System (Invitrogen). To examine the expression levels of LEDGF/p75 and p21 in ESCC cells, specific primer sets were designed and β‐actin was used as internal control. The primer sets, annealing temperature for amplification, and the length of PCR products are listed in Table 1. To ensure that the PCR reactions fall within the linear range of amplification, the proper numbers of cycling for the amplification of each control or target gene were examined (data not shown). For all reactions, total RNA extracted from the same cells without reverse transcription was used as negative control. The PCR mixture consisted of 10 mmol/L Tris–HCl pH 8.3, 50 mmol/L KCl, 1.5 mmol/L MgCl_2_, 0.01% gelatin, 200 pmol for each primer, 2 U Taq DNA polymerase (Promega), and 2 μL of sample cDNA. The resulting fragments were subjected to electrophoresis on a 1%‐2% agarose gel and visualized with ethidium bromide staining. All PCR reactions were repeated with reproducible results.

### 2.8 Patients and tissue samples

Total 218 paraffin‐embedded ESCC tissues were retrieved from the Pathology Department of Peking University Cancer Hospital & Institute from July 2003 to December 2007. The enrolled patients involved 156 men and 62 women, ages 38 to 79 years (median, 62 years), with stage I (n = 19), IIa (n = 79), IIb (n = 59), and III (n = 61) diseases according to the TNM system on the basis of AJCC classification.^29^ In all of these cases, the primary treatment was surgical and no patients had suffered from severe postoperative complications. All the specimens had been routinely formalin‐fixed, paraffin‐embedded, and serially sectioned at 4 μm in thickness. The clinical characteristics were collected from hospital records because all postoperative patients were routinely scheduled for a regular physical examination through visiting our hospital. This study was approved by both the Ethics and the Academic committees of Peking University Cancer Hospital & Institute, and informed consent was obtained from each subject.

### 2.9 DNA extraction and p21 genotyping

Formalin‐fixed and paraffin‐embedded tumor sections were histopathologically reviewed by two trained pathologists and the cancer tissues were separated using manual microdissection. Tissues were deparaffinized in xylene and then incubated overnight at 56°C in 50 μL of the digestion buffer containing 10 mmol/L Tris, pH 7.6, 100 mmol/L NaCl, 10 mmol/L EDTA, pH 8.0, 0.5% Tween 20, and 200 μg/mL proteinase K. The next day, proteinase K was inactivated by incubation of the samples at 100°C for 10 minutes. DNA samples were stored at −80°C until analysis. The rs2395655 variant genotype of the p21 promoter was determined by PCR direct sequencing and the primer sets are listed in Table 1. PCR was performed using 50 ng each sample DNA and negative controls (extracted slices of paraffin blocks containing no tissue) were included. The PCR products were subjected to direct sequencing using ABI 3700 DNA sequencer (PE Applied Biosystems, Norwalk, CT). For 15% samples, DNA extraction and PCR direct sequencing were repeated.

### 2.10 Immunohistochemistry (IHC) analysis

All tissues were stained using S‐P immunohistochemical method. Briefly, the slides were dewaxed in xylene, rehydrated in graded alcohols, and then treated with PBS containing 3% hydrogen dioxide to block endogenous peroxidase activity. After preincubating in 10% goat serum for nonspecific binding block, slides were then incubated overnight at 4°C with mouse anti‐human p21 protein (BD Biosciences; 1:500 dilution). After rinsing with 0.1% PBS‐T, sections were subsequently incubated with biotin‐conjugated IgG (Santa Cruz) with 1:10 000 dilution for 15 minutes at room temperature, followed by incubation with streptavidin‐peroxidase conjugate for 15 minutes. The signals were developed with DAB‐H_2_O_2_ solution. The slides were counterstained with 5% hematoxylin, and then examined by light microscopy. Sections without primary antibody treatment were used as negative control. Immunohistochemical evaluation of the p21 protein expression in ESCC specimens was based on the intensity and extent of nuclear reactivity. Moderate to strong p21 nuclear staining in ≥10% of tumor cells was defined as positive result. All ESCC sections were histopathologically reviewed by two trained pathologists.

### 2.11 Statistical analysis

SPSS 15.0 software (SPSS Inc, Chicago, IL) was used in determining statistical significance. The continuous variables from different groups were compared using *t* test. The associations of clinicopathologic characteristics and genetic variant with the p21 protein expression were tested by χ^2^ test. Disease‐free survival was defined as survival without the development of local recurrence or distance metastases. Univariate survival analysis was carried out by Kaplan–Meier method using log‐rank test. The Cox proportional hazards model was used for multivariate analysis. The variables in the multivariate analysis were age, sex, tumor location, tumor cell differentiation, TNM stage, p21 expression, and rs2395655 variant genotype. Values of *P *<* *0.05 were considered statistically significant.

## 3 RESULTS

### 3.1 Effects of polymorphisms rs3829963 and rs2395655 on transcriptional activity of the p21 promoter in ESCC cells

**Figure 1:**
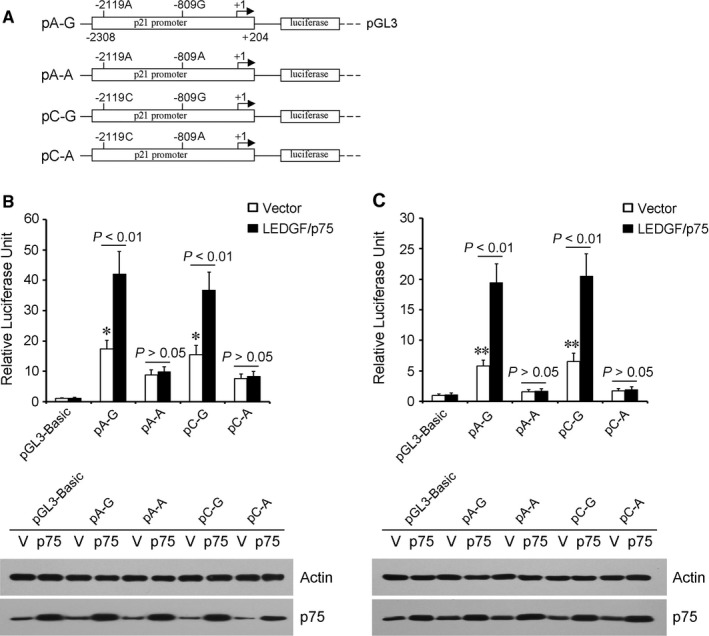
Reporter gene assay with constructs containing the pivotal region of the p21 promoter. (A) Schematic of four reporter gene constructs encompassing nucleotides −2308 to +204 of the p21 promoter, with sequences identical except for rs3829963 (–2119C/A) and rs2395655 (–809G/A) polymorphic sites. Luciferase expression in the four constructs in ESCC cells EC‐109 (B) and KYSE150 (C) were standardized by cotransfection with pRL‐SV40. LEDGF/p75 expression vector was cotransfected to evaluate its potential effect on the activity of the p21 promoter, using pcDNA3.0 empty vector as control. Fold increase was measured by defining the activity of the pGL3‐Basic vector and pcDNA3.0 empty vector as 1. The value of each reported construct was calculated as the mean fold increase ± SD from three independent experiments each performed in duplicate (top panel). LEDGF/p75 (p75 in brief) protein levels in EC‐109 and KYSE150 cells were examined by Western blot analysis (bottom panel). *P <* *0.05 and **P <* *0.01 vs rs2395655 A allele‐containing counterparts, respectively

To evaluate the effects of polymorphisms rs3829963 (–2119C/A) and rs2395655 (–809G/A) on transcriptional activity of the p21 promoter, reporter constructs were created with each combination of the two SNPs (Figure 1A). The results of luciferase assay showed that reporter gene expression driven by rs2395655 G allele‐containing promoters were about two and fourfold greater than A allele‐containing counterparts in EC‐109 (Figure 1B) and KYSE150 cells (Figure 1C), respectively. Ectopic LEDGF/p75 expression further increased the transcriptional activity of rs2395655 G allele‐containing p21 promoters by about two and three times in EC‐109 and KYSE150 cells, respectively. However, no significant difference between effects of rs3829963 C or A allele‐containing haplotypes on the p21 promoter activity was observed. Notably, ectopic LEDGF/p75 expression did not increase the transcriptional activity of rs2395655 A allele‐containing p21 promoters in both EC‐109 and KYSE150 cells. These results indicated that the p21 promoter with rs2395655 G allele displayed significantly higher transcriptional activity than rs2395655 A allele‐containing counterpart and LEDGF/p75 showed the ability of increasing transcriptional activity of the rs2395655 G allele‐containing p21 promoter in ESCC cells.

### 3.2 LEDGF/p75 was confirmed to bind rs2395655 of the p21 promoter by LEDGF/p75‐antibody EMSA and ChIP assay

**Figure 2:**
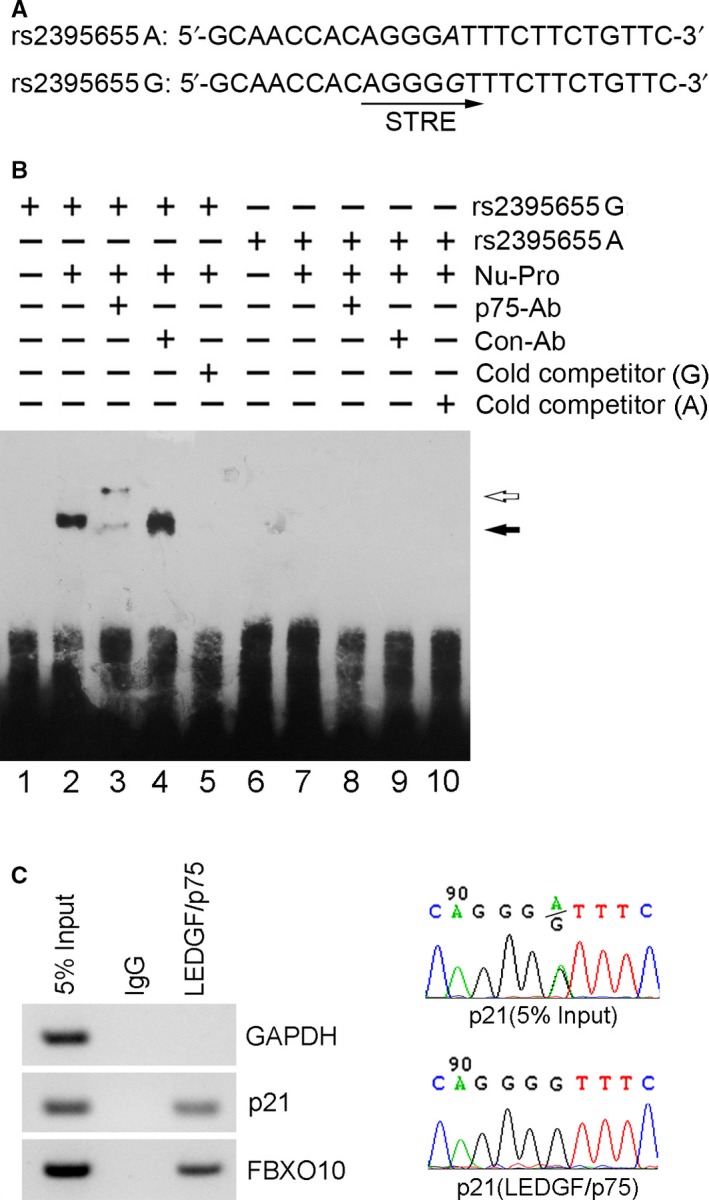
LEDGF/p75 interacted with human genomic DNA sequence containing rs2395655 G allele. (A) The rs2395655 A>G transition created one cis‐regulatory element, that is STRE element (AGGGGT, nucleotides −810 to −805), in the p21 promoter. Polymorphic sites of rs2395655 were italicized. (B) Nuclear extracts from KYSE150 cells were used for electrophoretic mobility shift assay (EMSA) with two biotin‐labeled probes carrying rs2395655 G and A allele, respectively. Anti‐LEDGF/p75 antibody (C‐16, 2 mg), IgG control antibody or a 400‐fold molar excess of cold probe was added to the binding reaction to examine the specificity of the protein‐DNA complex formation. The black and hollow arrows indicated the shift and supershift bands, respectively. (C) Chromatin immunoprecipitation was performed using anti‐LEDGF/p75 antibody (C‐16) and genomic DNA mixture of EC‐109 and KYSE410 cells, carrying rs2395655 GG and AA genotype, respectively. Human p21 region of interest was amplified and sequenced. Human GAPDH and FBXO10 loci were included as respective negative and positive LEDGF/p75 binding controls

Bioinformatic analysis using the TFSEARCH program predicted that polymorphism rs2395655 A>G transition created one *cis*‐regulatory element, that is STRE element (AGGGGT), comprising nucleotides −810 to −805 of the p21 promoter (Figure 2A). EMSA was performed to determine whether the different transcriptional activity of the rs2395655 G or A allele‐containing promoter is attributable to its binding capability to specific transcription factor(s). As shown in Figure 2B, a clear shift band was detected with the rs2395655 G probe (lane 2), which could be supershifted by specific anti‐LEDGF/p75 antibody (C‐16) (lane 3) but not by anti‐IgG antibody (lane 4). Further competition experiment with a 400‐fold molar excess of unlabeled rs2395655 G probe (lane 5) showed marked reduction in shift signal. In contrast, no positive binding reactions were observed with the rs2395655 A probe (lanes 7‐9). To further examine the allele specific binding ability of LEDGF/p75, we used the genomic DNA mixture obtained from EC‐109 and KYSE410 cells (carrying rs2395655 GG and AA genotype, respectively) and performed ChIP assay. The PCR amplification of input and IP products and subsequent sequencing results confirmed that LEDGF/p75 interacted with the p21 promoter region containing rs2395655 G allele (Figure 2C). These results suggested the potential binding capability of LEDGF/p75 with the STRE element implicated in rs2395655 G allele of the p21 promoter.

### 3.3 Effect of LEDGF/p75 on the p21 expression regulation in ESCC cells harboring rs2395655 GG or AA genotype

**Figure 3:**
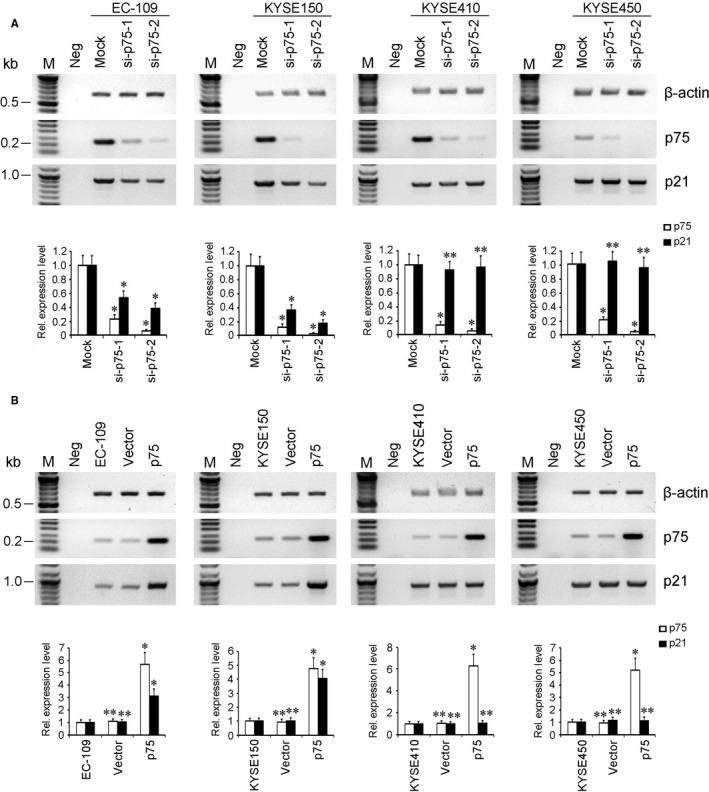
LEDGF/p75 regulated the expression of p21 gene in ESCC cells with rs2395655 GG genotype. (A) Specific small interference RNAs (si‐p75‐1 and si‐p75‐2)‐mediated depletion of LEDGF/p75 (p75 in brief) induced obviously down‐regulated expression of p21 mRNA in EC‐109 and KYSE150 cells carrying rs2395655 GG genotype but not KYSE410 and KYSE450 cells with rs2395655 AA genotype. (B) Ectopic expression of p75 apparently increased the expression level of p21 mRNA in EC‐109 and KYSE150 cells, while no change of the p21 expression level was detected in KYSE410 and KYSE450 cells. For specific siRNA transfection and ectopic expression of p75, the mismatched siRNA (mock) and empty vector transfections were used as control, respectively. The relative signal intensity of each target gene was quantified and normalized to internal control, β‐actin. The graph indicates the relative mRNA expression levels of p75 and p21 and the values are mean ± SD for three independent experiments. M, DNA marker. Neg, negative control. *P <* *0.01 and **P >* *0.05 vs controls, respectively

Four kinds of human ESCC cells carrying rs2395655 GG or AA genotype were used to examine the effect of RNA interference‐mediated depletion or ectopic expression of LEDGF/p75 on expression regulation of the p21 gene. Signal intensity scanning of RT‐PCR product bands of target and control genes was performed to determine the relative expression levels of target genes. As shown in Figure 3A, when compared with mock‐transfected control cells, transfection of specific siRNAs (si‐p75‐1 and si‐p75‐2) induced obviously down‐regulated expression of LEDGF/p75 messenger RNA (mRNA) in all four ESCC cells, with si‐p75‐2 displaying stronger effect than si‐p75‐1. Obviously down‐regulated expression level of p21 mRNA was also observed in EC‐109 and KYSE150 cells with rs2395655 GG genotype, but not in KYSE410 and KYSE450 cells with rs2395655 AA genotype. As expected, ectopic expression of LEDGF/p75 caused about three and fourfold increased expression level of p21 mRNA in EC‐109 and KYSE150 cells, respectively, while no change in p21 mRNA expression level was detected in both KYSE410 and KYSE450 cells following apparently up‐regulated expression of LEDGF/p75 (Figure 3B). These results supported our speculation that the interaction between LEDGF/p75 and the STRE element implicated in rs2395655 G allele played an important role in regulation of transcriptional activity of the p21 promoter.

### 3.4 Association of rs2395655 variant genotype with the p21 protein expression in ESCC tissues

**Figure 4:**
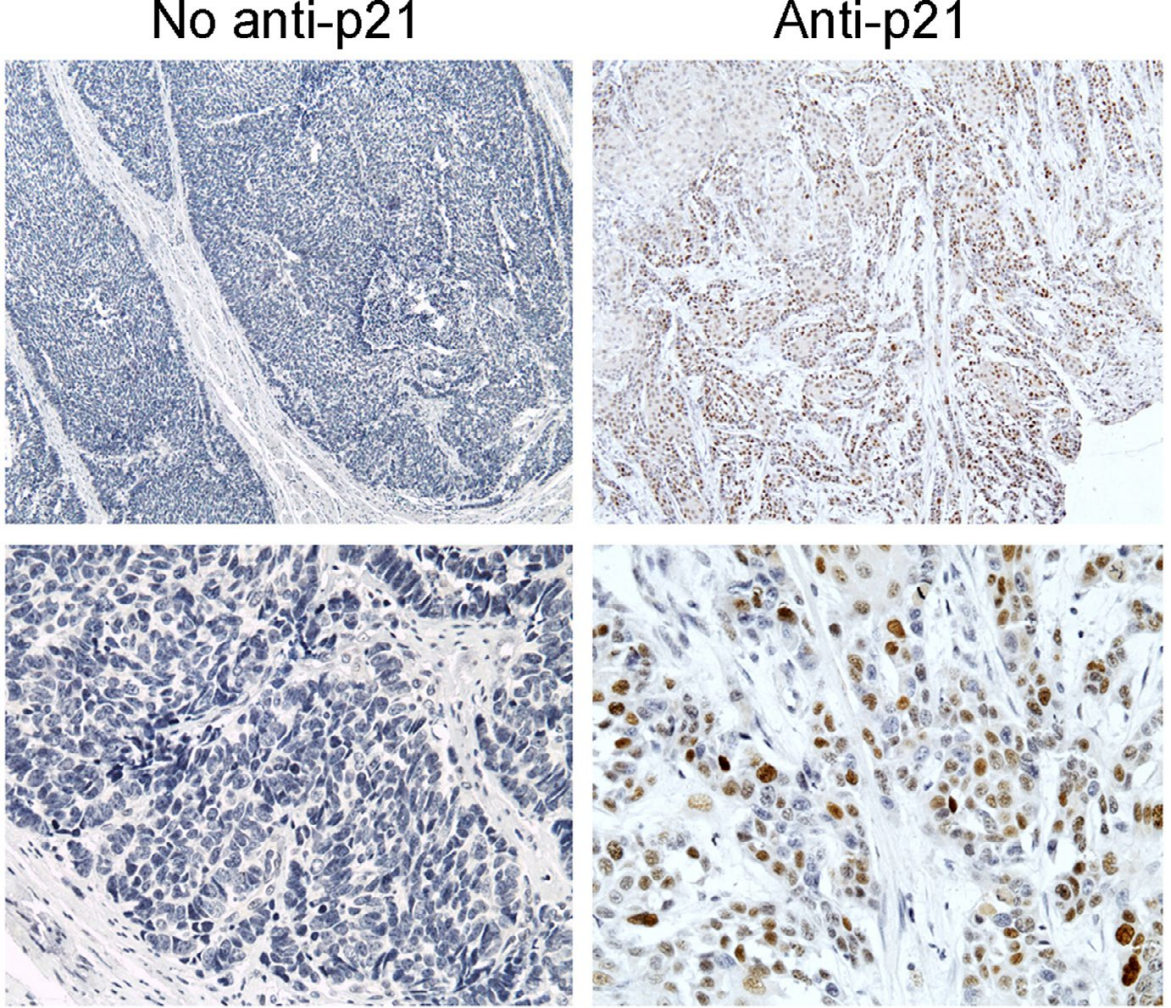
Immunohistochemistry staining of the p21 protein in ESCC tissues. No‐anti p21, negative control with primary antibody replaced by PBS. Anti‐p21, the brown signals represented positive staining for p21 protein and the staining was scored on a scale as indicated in the Materials and Methods. Positive cases were defined as those with moderate to strong p21 nuclear staining in ≥ 10% of tumor cells. Magnification, × 100 (top panel) and × 400 (bottom panel), respectively

The representative ESCC examples with p21 staining positive were shown in Figure 4. The brownish signals represented the positive staining and were found mainly in the nucleus of tumor cells. As shown in Table 2, total 78 (35.8%) out of 218 ESCC specimens were scored p21 staining positive. Chi‐squared test showed no significant correlation between the positive rate of p21 protein expression and clinical characteristics. The data were summarized in Table 2. The correlation between rs2395655 variant genotype and the p21 protein expression was further analyzed. PCR amplification of p21 rs2395655 locus was successful in 213 ESCC samples. Genotyping results showed that the frequencies for rs2395655 GG, AG, and AA were 29.6%, 45.1%, and 25.3%, respectively. We also performed genotyping of rs2395655 using DNAs from the blood samples of the same patient population and obtained the same results (data not shown). Statistical analysis revealed that rs2395655 GG genotype was significantly associated with up‐regulated p21 protein expression compared with rs2395655 AA or AG genotype (*P *=* *0.008).

**Table 2:** Associations of clinical characteristics and genetic factors with the p21 protein expression of ESCC patients (n = 218)

Clinical and genetic factors	p21 IHC no. (%)		*P* a Value
	Negative	Positive	
Age (y)			
≤60	67 (65.7)	35 (34.3)	0.672
>60	73 (62.9)	43 (37.1)	
Gender			
Male	101 (64.7)	55 (35.3)	0.798
Female	39 (62.9)	23 (37.1)	
Tumor location			
Upper	12 (56.7)	10 (43.3)	0.585
Middle	86 (66.7)	47 (33.3)	
Lower	42 (62.7)	21 (37.3)	
Tumor cell differentiation			
Well	49 (59.8)	33 (40.2)	0.547
Moderate	71 (66.4)	36 (33.6)	
Poor	20 (69.0)	9 (31.0)	
Tumor invasion (T)			
T1	13 (56.5)	10 (43.5)	0.482
T2	34 (59.6)	23 (40.4)	
T3	85 (66.4)	43 (33.6)	
T4	8 (80.0)	2 (20.0)	
Lymph nodes metastasis (N)			
N0	66 (58.6)	41 (41.4)	0.443
N1	74 (69.9)	37 (30.1)	
TNM stage			
I	10 (52.6)	9 (47.4)	0.261
IIa	48 (60.8)	31 (39.2)	
IIb	37 (62.7)	22 (37.3)	
III	45 (73.8)	16 (26.2)	
rs2395655 variant genotypes^b^			
GG	32 (50.8)	31 (49.2)	0.008
AA/AG	105 (70.0)	45 (30.0)	

a Two‐sided χ^2^ test.

b No information on some of the patients.

### 3.5 Associations of the p21 protein expression and rs2395655 variant genotype with disease‐free survival of ESCC patients

**Figure 5:**
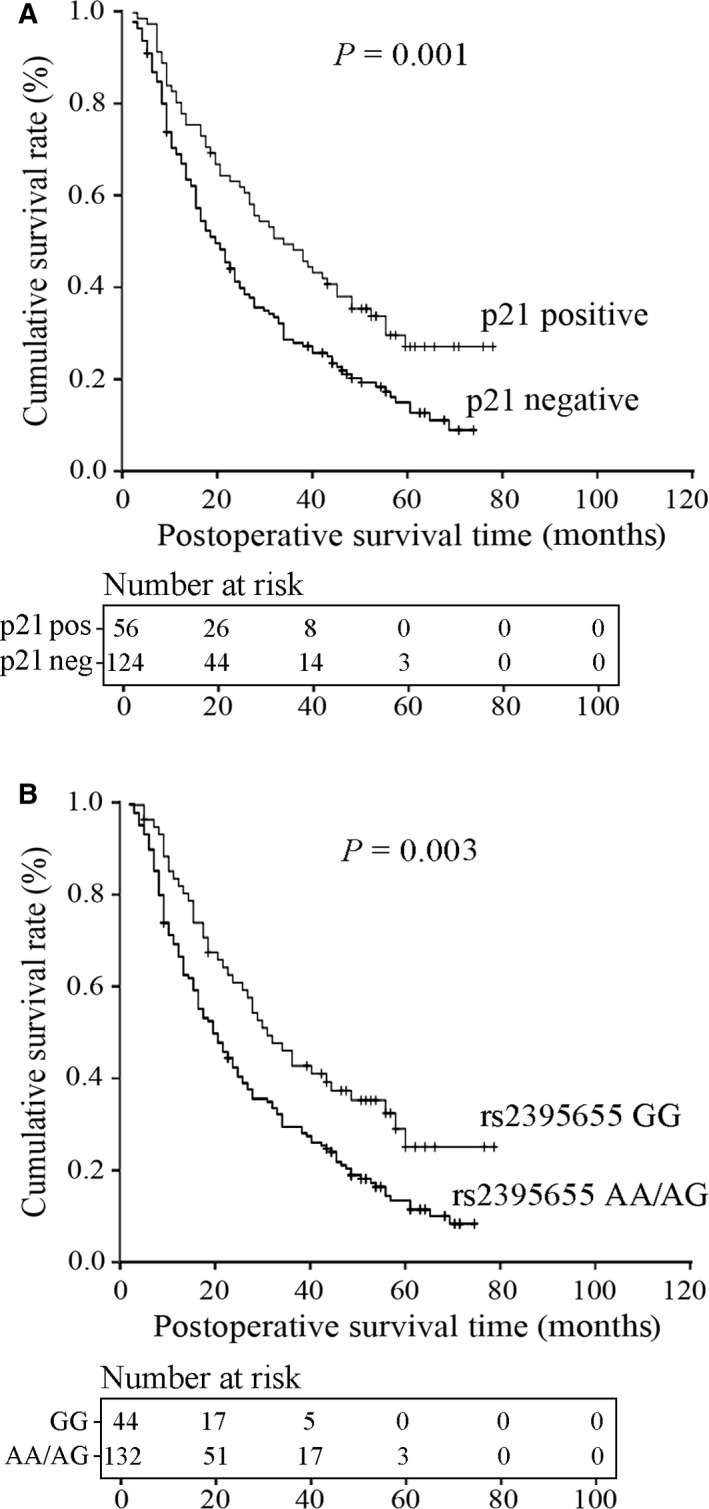
Kaplan–Meier survival estimation of 218 ESCC patients related to possible predictors. (A) Elevated p21 protein expression indicated better postoperative outcome for ESCC patients (32.0 and 19.0 months of median survival for p21‐positive and negative patients, respectively, *P *=* *0.001). (B) The median survival time of ESCC patients with rs2395655 GG genotype was significantly increased compared with rs2395655 AA or AG genotype (30.0 vs 20.0 months, *P *=* *0.003)

Univariate analysis using the log‐rank test revealed that the p21 protein expression and rs2395655 variant genotype were significantly associated with disease‐free survival time of ESCC patients. As shown in Figure 5A, the median disease‐free survival time for p21‐positive patients was obviously increased than p21‐negative patients (32.0 ± 4.8 vs 19.0 ± 2.1 months, *P *=* *0.001). Notably, ESCC patients carrying rs2395655 GG genotype showed better postoperative outcome than patients with rs2395655 AA or AG genotype (30.0 ± 3.9 vs 20.0 ± 1.8 months, *P *=* *0.003) (Figure 5B). Furthermore Cox multivariate analysis supported the positive prognostic roles of up‐regulated p21 protein expression and rs2395655 GG genotype in this series of 218 ESCC patients (*P *=* *0.008 and 0.025, respectively), as shown in Table 3.

**Table 3:** Independent predictors of disease‐free survival time in multivariate analysis in ESCC patients (n = 218)

Variables	Hazard ratio (95% confidence interval)	*P* a
TNM stage		
Stage IIb/III/IV vs stage I/IIa	2.005 (1.454‐2.765)	0.000
p21 expression		
Positive vs negative	0.645 (0.466‐0.893)	0.008
rs2395655 variant genotypes^b^		
GG vs AA/AG	0.666 (0.467‐0.950)	0.025

a The Cox proportional hazards model with a stepwise procedure.

b No information on some of the patients.

## 4 DISCUSSION

p21 is a principal mediator of cell cycle arrest in response to DNA damage and its expression is regulated by a complex cellular signaling pathway in which many nuclear proteins, including p53, E2F, Ap‐1, Ap‐2, c‐Jun, et al., interact with *cis*‐acting elements in the p21 promoter region and regulate p21 gene transcription.^3^, ^4^, ^14^, ^15^, ^16^, ^17^, ^18^, ^19^, ^20^, ^21^ Naturally occurring SNPs in the p21 promoter may therefore have impact on gene transcription by altering the binding capability of the promoter with certain nuclear proteins.

LEDGF/p75 is a survival factor that enhances growth and resistance to cell death induced by various environmental stress signals and regulates downstream response pathways by enhancing expression through binding to specific *cis*‐acting elements, namely, STRE and HSE (heat shock element), located in the promoter region of numerous genes, including antioxidant protein 2, Hsp27, alphaB‐crystallin, and VEGF‐C.^24^, ^30^, ^31^ In addition, LEDGF/p75 has emerged as an important cellular cofactor implicated in multiple pathways affecting cancer progression. LEDGF/p75 was identified to be overexpressed in several human tumor types, including prostate, colon, thyroid, liver, uterine, and breast cancers.^32^ It was revealed that alphaB‐crystallin, a target of LEDGF/p75, was expressed in basal‐like tumors and predicted poor survival in breast cancer patients.^33^ Increased expression of LEDGF/p75 was identified in blasts from chemotherapy resistant human acute myelogenic leukemia patients and demonstrated to protect leukemia cells from apoptosis.^34^ LEDGF/p75 was reported to tether the mixed‐lineage leukemia (MLL1) protein complex to chromatin and promote development of MLL leukemia.^35^ LEDGF/p75 was also found to bind to a regulatory region of FBXO10 and increase expression during conditions favoring carcinogenesis.^36^ LEDGF/p75:JPO2 protein complex was identified to be critical modulators of PI3K/AKT signaling and metastasis in medulloblastoma.^27^ It was recently demonstrated that LEDGF/p75 played an important role in breast cancer tumorigenicity by promoting the expression of genes controlling the cell cycle and tumor metastasis.^37^

In this study, two SNPs, that is rs3829963 (−2119C/A) and rs2395655 (−809G/A), were evaluated for their effects on the p21 transcriptional activity in ESCC cells. Bioinformatic analysis showed that rs2395655 A>G transition introduced one *cis*‐regulatory element, that is STRE element, into the p21 promoter region. Reporter constructs encompassing nucleotides −2308 to +204 of the p21 promoter with each combination of the above two SNPs were established for luciferase assay and the results showed that polymorphism rs2395655 but not rs3829963 influenced the p21 promoter activity in the context of the 2‐site haplotypes. Moreover, the p21 promoter containing rs2395655 G allele displayed significantly higher transcriptional activity than A allele‐containing counterpart, especially with ectopic LEDGF/p75 expression in ESCC cells. Furthermore, gel shift assay using the rs2395655 G or A allele‐containing probe and chromatin immunoprecipitation assay with specific anti‐LEDGF/p75 antibody indicated the potential binding activity of LEDGF/p75 with the STRE element implicated in rs2395655 G allele of the p21 promoter.

Next, four human ESCC cells with rs2395655 GG or AA genotype were used to examine the potential role of LEDGF/p75 and polymorphism rs2395655 in regulation of transcriptional activity of the p21 promoter. Specific RNA interference‐mediated depletion or ectopic expression of LEDGF/p75 caused obviously down‐ or up‐regulated expression level of the p21 mRNA in cells harboring rs2395655 GG genotype (EC‐109 and KYSE150) but not cells with rs2395655 AA genotype (KYSE410 and KYSE450). Additionally, si‐p75‐2 played a stronger role in down‐regulating the expression levels of both LEDGF/p75 and p21 in comparison with si‐p75‐1. These results were consistent with Singh DK's findings that p21 expression was obviously down‐regulated in LEDGF/p75‐depleted breast cancer cells.^37^ Our findings strongly supported the idea that LEDGF/p75 regulated p21 expression in ESCC cells through interacting with STRE element implicated in rs2395655 G allele.

Due to the regulatory effect of polymorphism rs2395655 on the p21 promoter activity in ESCC cells, we further performed immunohistochemical evaluation of the p21 protein expression in total 218 ESCC tissues and investigated the association of rs2395655 variant genotype with the p21 protein expression. Chi‐squared test showed significantly elevated p21 protein expression in ESCC tissues with rs2395655 GG genotype than tissues with rs2395655 AA or AG genotype (*P *=* *0.008). Moreover, the associations of the p21 protein expression and rs2395655 variant genotype with disease‐free survival time were evaluated in this ESCC population. Both univariate analysis and Cox multivariate analysis showed better postoperative outcome for p21‐positive ESCC patients than p21‐negative patients (*P *=* *0.001 and 0.008, respectively). Our results were consistent with those reports implying the positive prognostic role of elevated p21 expression in ESCC.^7^, ^8^, ^10^, ^11^, ^12^, ^13^ However, several other reports suggested an adverse prognostic role of up‐regulated p21 expression for ESCC patients.^5^, ^6^, ^9^ The inconsistency among these studies is most likely due to inadequate study design, limited populations with different geographic locations, or even different esophageal cancer types. Notably, univariate analysis revealed that the median survival time of ESCC patients with rs2395655 GG genotype was much longer than patients carrying rs2395655 AA or AG genotype (30.0 vs 20.0 months, *P *=* *0.003). Cox multivariate analysis further indicated a positive role of rs2395655 GG genotype in predicting the postoperative outcome of ESCC patients (*P *=* *0.025). These results of survival analysis were supported by the research findings of Ma H and his colleagues that p21 rs2395655 AA genotype was significantly associated with the increased risk of cancer death of non‐small cell lung cancer (adjusted HR = 1.38, 95% CI = 1.07‐1.78).^38^

Taken together, we demonstrated for the first time that LEDGF/p75 binds to STRE element implicated in polymorphism rs2395655 and regulates the p21 gene expression in ESCC cells and tissues. Our results suggested that the elevated p21 protein expression and rs2395655 GG genotype may serve as positive prognostic factors for ESCC patients. These findings further revealed the complicated roles of LEDGF/p75 as a survival factor and a cancer‐related protein, and provided a rationale for ongoing studies aimed at understanding the stress survival activity and the critical roles of LEDGF/p75 in specific human cancers.

## CONFLICT OF INTEREST

The authors have no conflict of interest to declare.
